# Pre-exposure prophylaxis as an opportunity for engagement in HIV prevention among South African adolescents

**DOI:** 10.1080/17290376.2021.2016479

**Published:** 2022-02-08

**Authors:** Ashleigh LoVette, Caroline Kuo, Danielle Giovenco, Jacqueline Hoare, Kristen Underhill, Don Operario

**Affiliations:** a Department of Behavioral and Social Sciences, Brown University School of Public Health, Providence, RI, USA; b Department of Psychiatry and Mental Health, University of Cape Town, Cape Town, SA, USA; c Department of Epidemiology, University of North Carolina at Chapel Hill, Chapel Hill, NC, USA; d Columbia Law School, New York, NY, USA

**Keywords:** Biomedical, healthcare, prevention, sub-Saharan Africa, youth

## Abstract

Pre-exposure prophylaxis (PrEP) offers a potential biomedical strategy to reduce HIV incidence among adolescent populations disproportionately affected by HIV. There is limited evidence on the social and clinical implications, including engagement in HIV prevention efforts, of PrEP for South African adolescents, who face high HIV risk. We conducted a mixed-methods study in Western Cape, South Africa from 2015 to 2016. Adolescents (*N* = 35) aged 16–17 and clinical service providers working with adolescents (*N* = 25) were recruited from community and clinic settings. Adolescents and service providers completed a survey about their overall perceptions of PrEP and completed interviews guided by semi-structured protocols. We performed descriptive analysis of quantitative data using SPSS and thematic analysis of qualitative data using NVivo. The majority of adolescents endorsed future PrEP use for themselves and partners, and all clinical service providers endorsed future PrEP use for sexually active adolescents. Both adolescents and service providers identified PrEP as an opportunity to engage youth as active participants in HIV prevention. Service providers also viewed PrEP as a potential mechanism for shifting life trajectories. Findings from this study enhance our understanding of the considerations needed to engage adolescents and clinical service providers in the roll-out of oral PrEP in South Africa.

## Introduction

Young people across the world are disproportionately infected with HIV, accounting for 40 percent of all new infections (UNAIDS, 2012a; 2014). In South Africa, home to one of the largest epidemic, adolescents are disproportionately at risk for HIV (Cowan & Pettifor, 2009; Harrison, Colvin, Kuo, Swartz, & Lurie, 2015). Pre-exposure prophylaxis (PrEP) offers an important expansion to the HIV prevention toolbox for adolescents and young people, who are a priority population for HIV prevention (UNAIDSb, 2012). However, the under-representation of adolescents in biomedical HIV prevention research, especially those under 18 years of age, remains a significant challenge to integrating biomedical strategies, such as oral PrEP, into effective combination prevention packages for this important risk group (MacQueen & Karim, 2007; Pettifor, Stoner, Pike, & Bekker, 2018). If PrEP is to be fully integrated into combination HIV prevention efforts, we urgently need to understand age- and developmentally-specific social and behavioural factors that influence acceptance, adoption and widespread use of oral PrEP among adolescents in generalised epidemic settings, such as South Africa (Hosek et al., 2016).

An understanding of adolescent-specific social and behavioural factors that will shape the engagement of youth as active participants in roll-out and uptake of PrEP is crucial for achieving the maximum prevention potential of PrEP. Research can be an important part of this engagement process, by involving the perspectives of adolescents in the development, implementation, and evaluation of HIV prevention programming. Engaging adolescents in prevention efforts can also facilitate the design and implementation of adolescent-friendly health care (UNAIDS, 2016; WHO, 2015). Through active participation, youth can become partners and advocates for their sexual and reproductive health, as well as the health of their peers. This transformative process can be particularly useful when rolling out new biomedical HIV prevention strategies, such as oral PrEP.

Adolescents’ engagement in PrEP care is likely to be different from adults because of unique social and developmental characteristics within this age group. Adolescence marks a time of many life changes relating to sexual health and relationships that may uniquely affect the acceptability of PrEP and willingness to use or support partner PrEP use (Kapogiannis, Nelson, Siberry, Lee, & Hazra, 2018). For example, this population is likely initiating their first romantic and sexual relationships, which can present particular challenges around decisions to use prevention strategies such as PrEP. Furthermore, adolescents are more likely to be in school, and to be living with parents or caregivers, and less likely to have independent access to health services. This dependence upon adults can present some challenges for uptake of PrEP among this population. This age group may also have observed generational effects of the HIV epidemic, including familial experiences with HIV-related illness and death, as well as treatment adherence (Cluver & Operario, 2008; Edström & Khan, 2009). These experiences are especially relevant in generalised epidemic settings such as South Africa, and also shape adolescents’ engagement in ongoing HIV prevention efforts (Operario, Underhill, Chuong, & Cluver, 2011; Sherr et al., 2014). It is vital to consider all of these age-specific and developmental influences when engaging adolescents in HIV prevention efforts.

To fully understand the behavioural and social implications for adolescent PrEP usage, it is also necessary to explore clinical service providers’ understandings of PrEP. Their opinions around the acceptability of PrEP for adolescents, willingness to prescribe to this population, and perspectives on the potential behavioural consequences of PrEP on adolescent health can directly inform the implementation of this HIV prevention care in an adolescent-centred manner. Recent research has also highlighted the important role of clinicians and other health service providers as gateways into both preventative care and treatment (Calabrese, Krakower, & Mayer, 2017; Marcus et al., 2018). This study builds our understanding of the behavioural, social and clinical considerations needed to engage both adolescents and service providers in the roll-out of oral PrEP in South Africa, a priority setting for the global HIV epidemic.

## Methods

We collected quantitative and qualitative data in Western Cape, South Africa from 2015 to 2016. First, we conducted focus group discussions with HIV-positive adolescents and HIV-negative adolescents to obtain preliminary insights into some of the salient community perspectives on PrEP use with adolescents. Full description of the methods and focus group findings is described elsewhere (Giovenco, Kuo, Underhill, Hoare, & Operario, 2018). Following the completion of focus groups, we conducted in-depth individual interviews to explore more personal perspectives with *N* = 35 adolescents, living with and without HIV, who previously took part in focus groups and who declared interest in further participation. Additionally, we conducted *N* = 25 individual interviews with clinical service providers who work with adolescents to explore perceptions from professionals who are likely to counsel and prescribe PrEP to adolescents in the future. This paper purposively uses data from the individual interviews (*N* = 60) of adolescents and clinical service providers, as both types of participants used one-on-one interviews to share personal experiences and perspectives of future engagement with oral PrEP.

Adolescents living with HIV were recruited from adolescent HIV treatment clinics and adolescents living without HIV were recruited via door-to-door community sampling. Eligibility criteria were assessed at initial in-person screening. Adolescents living with HIV were eligible if they met the following inclusion criteria: (1) 16–17 years of age; (2) self-reported HIV-positive status and (3) comfort discussing their HIV status in the presence of others. Adolescents living without HIV were eligible if they met the following inclusion criteria: (1) 16–17 years of age and (2) self-reported HIV-negative status. Trained research staff obtained written parental consent and adolescent assent. Participants who were unable to give informed assent and/or whose parents/caregivers did not provide consent were excluded. Interviews were conducted in isiXhosa or English based on participant preference.

Prior to each interview, facilitators provided adolescent participants with a verbal description of oral PrEP, including information about its efficacy, side effects, HIV testing requirements and the importance of adherence. They were informed that PrEP research to date had involved only adults, and that research from adolescents was needed. Adolescent participants then completed a brief ACASI-administered survey that included questions about lifetime sexual behaviours and condom use, willingness to use PrEP, willingness to support a partner’s use of PrEP, and sociodemographic characteristics. Interviews each lasted approximately 1–1.5 h and were digitally audiotaped. Adolescent participants received 150 Rand (approximately 12 USD) for completing focus group and/or individual interviews.

Clinical service providers were eligible if they were: (1) 18 years or older and (2) had 3 or more years of experience providing services to adolescents. An initial seed-pool of participants was generated in consultation with the study team, and these initial participants referred professional peers to the study. Following written consent, service providers were given a description of oral PrEP and a brief quantitative survey was administered. Then, one-on-one interviews were conducted in isiXhosa or English. Interviews lasted approximately 1–1.5 h and were digitally audiotaped. Interviews were guided by semi-structured protocols exploring willingness to prescribe PrEP and perceptions around its influence in the care they provide. Each participant provider received 300 Rand (approximately 20 USD).

We conducted descriptive analysis of quantitative data using SPSS (IBM, 2016) and thematic analysis of qualitative data using NVivo (QSR International, 2012). Data were transcribed verbatim and all transcripts were double coded. Data were analysed using open-coding, axial coding, and coding of marginal remarks and comparisons (Strauss & Corbin, 1998). Common words, phrases, sentences, and ideas were clustered to develop a codebook. These pieces of text were compiled across all interviews under specific codes and sub-codes. Meaning from these codes was formulated to produce themes presented below. All study procedures were approved by ethical review committees at Brown University (Protocol #1207000666) and University of Cape Town (Protocol #HREC 072/2013).

## Results

### Participant characteristics and quantitative data

Participant characteristics are outlined separately for adolescents (*N* = 35) and providers (*N* = 25). As detailed in Table 1, there were slightly more female adolescent participants than male adolescent participants as well as more participants aged 16 than aged 17 years. A significant majority (80%) of adolescent participants indicated they were sexually active. Table 2 includes information about clinical service providers. The majority of clinical service providers were female with an average age of 41.27 years. Other languages spoken included isiZulu and French, and other provider types included psychologists and patient support managers.

**Table 1.** Participant characteristics among adolescents (*N* = 35)

	Frequency (%)
Sex Female Male	20 (57.1) 15 (42.9)
Age 16 17	21 (60.0) 14 (40.0)
Race/ethnicity Black African White	34 (97.1) 1 (2.9)
Primary language isiXhosa English Afrikaans	32 (91.4) 2 (5.7) 1 (2.9)
HIV Status Living with HIV Living without HIV	10 (28.6) 25 (71.4)
Sexual behaviour Sexually active Sexual orientation *Straight Bisexual*	28 (80) 33 (94.3) 2 (5.7)

**Table 2.** Participant characteristics among clinical service providers (*N* = 25).

	Frequency (%)
Sex Female Male	24 (96) 1 (4)
Race/ethnicity Black African White Coloured Other	12 (48) 10 (40) 1 (4) 2 (8)
Primary language English isiXhosa Afrikaans Other	13 (52) 9 (36) 1 (4) 2 (8)
Education High School Bachelors Masters, Doctoral or Specialised Training	12 (48) 3 (12) 10 (40)
Service provider type Doctors Nurses Counsellors Other	10 (40) 4 (16) 7 (28) 4 (16)

After learning basic information about oral PrEP at the beginning of the study, the majority (83%) of interviewed adolescents reported interest in using PrEP if they had a partner who was HIV-positive or of unknown status. Also, a majority (86%) of adolescents living with HIV would support the use of PrEP by an HIV-negative partner. All (100%) clinical service providers endorsed future prescription of PrEP for sexually-active adolescents. Together, these quantitative results demonstrated a strong interest around adolescent PrEP usage among adolescents and clinical service providers, which could then be explored in-depth through qualitative interviews.

### Qualitative data

While the quantitative results indicated support of future oral PrEP usage by adolescents, two main qualitative themes provided context for this support and also highlighted potential behavioural and social implications of PrEP usage among adolescents. First, both adolescents and clinical service providers identified PrEP as a unique opportunity to engage youth as active, rather than passive, participants in HIV prevention and overall health promotion efforts. Second, service providers viewed PrEP as a mechanism for changing adolescents’ life trajectories, and as a tool to promote health and well-being in high-risk adolescent populations and their partners.

#### PrEP as prevention tool to facilitate engagement in health promotion efforts: adolescent perspectives

Several adolescents viewed potential conversations about PrEP usage as an invitation to reflect on the larger context of prevention efforts, particularly when it came to decisions around sexual health and relationships. This included adolescents envisioning their relationships as one aspect within the dynamic context of their lives. In the excerpt below, a participant considered the role of PrEP usage beyond the context of a current sexual partnership, and reflected on PrEP as a way to protect themselves as well as future partners.

Because after all, I’m still young. Perhaps this person I’m dating … we’re still gonna part ways eventually. So, I have to protect myself so that any other person I get together can be able to be safe. *Adolescent living without HIV*

Participants also shared how they would engage with their parents and caregivers in conversations about oral PrEP, with emphasis on how it can enhance safety in an unpredictable and hazardous environment. For example, one adolescent used a metaphor of ‘defensive-driving’ as a way to convey the necessity of PrEP usage among adolescents within the context of high risk, and another noted how referencing PrEP can help facilitate discussions between adolescents and their parents about sexual choice and safety.

The child can say that, “Mom, the reason for me to use [PrEP] is this. It is going to protect me from stuff such – I don’t know - Like, there are car accidents, you don’t know what you’re going to be [faced with], although you [are] on the road. So, it’s better to be safe – the main thing.” – Adolescent living without HIV

Maybe she might understand. Because she understands if you tell her, “[Well], I’m using this pill to protect myself from things out there.” Inside she knows that, ‘Ok, the daughter/girl [has reached] the stage now. – Adolescent living with HIV

The perceptions of adolescents living with HIV were shaped by their lived experiences with their attitudes towards PrEP ranging from hopeful to hesitant. While several adolescents discussed a sense of agency around future oral PrEP use, others noted that this engagement could be limited by judgement from peers.

Like, if they – they take the pill and maybe, like, they have their friends over – and then they see, like, the time – they will feel ashamed to, like, to take this pills and then to drink it front of their friends. Because their friends – they think that they will – they will judge them. – Adolescent living with HIV

Overall, results from adolescents revealed how adolescents perceived that PrEP availability could serve as tool in managing uncertainties about their future health risks due to the high rates of violence and HIV infection. A majority indicated a strong interest in taking steps to protect this future, and many envisioned the need for bringing parents into conversations about PrEP.

#### PrEP as prevention tool to facilitate engagement in health promotion efforts: clinical service provider perspectives

Clinical service providers viewed discussions of PrEP usage with adolescents as an opportunity to help them foster independence and responsibility for their health, as well as support decision-making processes that are vital for healthy adolescent development. In this way, the providers acknowledged the potential of biomedical interventions, like PrEP, to empower adolescents to participate in the learning and decisions around their own sexual health.

[PrEP] is empowering that teenager in responsibility and trust. Trust that they then engender in their sexual partner. Also in how the family may see them in their responsibility in their behaviour. If they check that anybody that they have, who’s gonna be a potential partner, that they don’t just take the usual precautions but they also check that they are on PrEP, then it means that they’re taking responsibility and growing up, in some ways. – Doctor

Results also emphasised that conversations between adolescents and providers about oral PrEP usage could foster the mastery of skills that are key parts of adolescent development. The potential of PrEP to help girls and young women specifically engage with preventative health efforts was highlighted below by a doctor.

I think [PrEP] empowers the girl, the woman who doesn’t need to negotiate it with a partner who is abusive, whether it’s sexually, physically, emotionally abusive. I think for someone to have the option of themselves making the decision to be able to protect themselves, to take responsibility, have power over decisions that will protect their health. Then it’s not at the hands of the other partner in the relationship. I think that’s enormously empowering. – Doctor

Through a lens of empowerment, clinical service providers recognised that PrEP has the potential to redistribute agency to girls and young women, who historically have faced limited sexual agency and decision making power due to social and structural inequities.

#### PrEP as a prevention tool to shift life trajectories

With years of experience working with adolescent patients in this HIV-endemic setting, service providers recognised the potential of PrEP to shift important aspects of adolescents’ lives. In particular, PrEP was viewed as a dynamic tool to assist adolescents during their developmental journey into adulthood.

This is the time for us to give them information, that PrEP is there and it is going to help them to use it so that they don’t get infected. So the very thing that made me happy is that, there is something that is going to protect our people, especially the youth, because I am very much concerned about them—because they are our future, you see. - Counsellor

One nurse acknowledged that while adolescence is a period of rapid development, growth, and experiences that could come with great risk within this context, oral PrEP usage could support teens through this tumultuous time and towards a bright future.

I think it [PrEP] is going to help a lot because they are - we are much more worried about the children when they’re still growing up. The children have a bright future. Because now sex is everywhere. Children want to experience everything these days. I think we can also support them by giving them the right stuff to do. - Nurse

Another clinical service provider discussed using PrEP as a catalyst for young people to reflect upon their past, present, and future life trajectories as they navigate young adulthood.

I just thought of an exercise that we used to do around when we used to do training on teenage pregnancy and stuff. We’d ask the young people to draw a map of the next five years. Draw your road and then mark down important points along the road, or even for the next two years or whatever. I want to do this. I want to do that. Then they imagine it and then you tell them, “Imagine at this point you fall pregnant. What’s going to happen? How’s your road going to change? Are you going to be able to do those things?” Maybe they just realize, okay, they realize the impact that it could have on what they want to do. Maybe something similar like that around PrEP. – Other (Patient Support Manager)

Several service providers recognised the relevance of PrEP for improving the sexual health and relationship futures for adolescents living with HIV, who may otherwise feel disempowered or restricted in their sexual partnerships and relationship futures.

A lot of them have real concerns about adult relationships and about marriage and about settling down, and they've just—they feel like that might not be available to them and I think they might find it quite an encouraging and hopeful thing that they—that there would be something they could give to their partners to protect them. – Doctor

Results highlighted the potential of PrEP as a prevention tool that may also support healthy and life-changing relationship behaviours for adolescents living with HIV, such as disclosure to partners.

## Discussion

Whereas the importance of oral PrEP as a potential tool for reducing HIV transmission has been well described, findings from this mixed-methods study provide additional insights into the role of PrEP as a potential source to foster personal agency and enhance sexual health communication with partners and providers among adolescents in HIV-endemic settings such as urban South Africa (Mullins & Lehmann, 2018; Rotheram-Borus, Davis, & Rezai, 2018). Findings also indicate that the successful implementation of PrEP as a biobehavioural prevention method has the potential to catalyse other prevention behaviours that can promote sexual and reproductive health during this developmental window into adulthood. Future programming that offers oral PrEP may want to capitalise on these prevention opportunities by including information and support for other cascading prevention behaviours (e.g. testing, dual protection, disclosure) in addition to information regarding the uptake of PrEP.

This study also reveals the utility of examining adolescent perspectives and the willingness of many adolescents to contribute their perspectives on biomedical HIV prevention. To create safe, effective, and acceptable HIV prevention tools to address the health needs of South African adolescents, they need to fully participate in the HIV/STI research process (Rosenthal, Morris, Hoffman, & Zimet, 2018). A synthesis report from WHO notes that empowering young people can have positive effects on health outcomes (Wallerstein, 2006). Engaging adolescents and young people in strategies to support scale-up of novel prevention strategies, such as PrEP, is one such strategy to empower youth.

Given that adolescents and young people face disproportionate risk for HIV infection, these findings are important as policy decisions are made around the disbursement of anti-retroviral drugs for both prevention and treatment to priority populations. The research and policy landscape around PrEP in South Africa is fast changing, particularly among adolescents. As PrEP usage continues to increase globally, future research should explore ways to engage adolescents in HIV prevention efforts so that perspectives reflecting their specific developmental needs are integrated into approaches at each stage of the PrEP care continuum (Nunn et al., 2017). This includes having adolescents weigh in on the design of biomedical and combination prevention programming prior to roll-out, as well as carefully considering the developmental implications for each stage of the continuum. For example, concepts such as peer influence and experimentation may be of particular relevance for promoting uptake of PrEP among adolescents.

There are several limitations to this research. As is consistent with other studies using qualitative methods, the small sample size may limit generalisability to other populations. The authors also acknowledge that these results may not be representative of all clinical service providers. Despite these potential concerns regarding the generalisability of results, this research provides valuable insight into motivations and decision-making of service providers of adolescent care within an HIV endemic setting. Studies such as these can also identify potential issues, or areas of concern, that could be mitigated before roll-out among adolescents in settings where they are designated as key priority populations. Finally, it is important to note that while oral PrEP was not available at the time of interviews, it is now available. Despite this temporal limitation, this study demonstrates the importance of engaging adolescents throughout the research process, a practice that is key for translational biomedical research.

## Conclusion

Further research is needed to understand support for PrEP use and PrEP’s potential behavioural impact not only among HIV-negative adolescents but also among their future partners given the high probability of adolescents engaging in serodiscordant relationships or in relationships with partners of unknown status. Additional research should also explore the complex legal and policy landscape around PrEP access in different country settings. In South Africa, the cost of obtaining PrEP will be a critical consideration for implementation with adolescents. The legal context can have important implications for access including whether adolescents have autonomy around whether or not to take PrEP or seek parental/guardian consent (Moore, Paul, McGuire, & Majumder, 2016). Through this future work, researchers and clinical service providers can continue to invest in adolescents’ health and well-being to ensure a generation of healthy adults for tomorrow (Sawyer et al., 2012; Viner & Barker, 2005).
